# Conservative Management Versus Arthroscopic Repair for Symptomatic Partial-Thickness Supraspinatus Tears: A Prospective Comparative Analytical Study

**DOI:** 10.7759/cureus.102351

**Published:** 2026-01-26

**Authors:** Prateek Khanna, Jagdeep Rehncy, Daljinder Singh, Nirnhai Singh, Saurav Soni, Girish Sahni, Sachin Goel

**Affiliations:** 1 Orthopaedics, Government Medical College, Patiala, IND; 2 Ophthalmology, Civil Hospital Badal, Muktsar, Punjab, IND

**Keywords:** arthroscopic repair, conservative management, partial-thickness tears, physiotherapy, rotator cuff tear

## Abstract

Introduction

The optimal management of symptomatic partial-thickness rotator cuff tears (Ellman Grade II and III) remains controversial. While meta-analyses exist for full-thickness tears, prospective comparative data specifically for these intermediate lesions is limited. This study compared the functional outcomes of structured conservative rehabilitation versus arthroscopic repair to determine therapeutic efficacy.

Methods

A prospective comparative analytical study was conducted involving 50 patients with MRI-confirmed symptomatic partial-thickness supraspinatus tears. Patients were allocated to either conservative management (n=25) involving a supervised FITT-based rehabilitation protocol or arthroscopic repair (n=25) based on shared decision-making. Functional outcomes were assessed using the Constant-Murley Score (CMS) and Visual Analog Scale (VAS) at baseline, 1 month, and 6 months.

Results

Baseline characteristics were well-matched. At six months, both groups demonstrated significant functional improvement, with no statistically significant difference in final CMS (conservative: 75.7 ± 5.9 vs. surgical: 75.0 ± 6.7; p=0.723). The conservative group demonstrated a significant early functional advantage at 1 month (p<0.001) due to the avoidance of postoperative immobilization. While the surgical group achieved statistically lower absolute pain scores at 6 months (p=0.007), the mean difference (0.40 points) did not exceed the Minimal Clinically Important Difference (MCID).

Conclusion

Structured conservative management yields short-term functional outcomes equivalent to arthroscopic repair for symptomatic partial-thickness supraspinatus tears, with the added benefit of faster early recovery. These findings support conservative care as a robust first-line strategy. Limitations include the non-randomized design and short-term follow-up, necessitating future longitudinal research to assess tear progression.

## Introduction

Rotator cuff pathology constitutes a predominant source of shoulder morbidity in the adult population, accounting for a substantial economic and clinical burden globally. While the management of full-thickness tears is relatively well-defined, partial-thickness supraspinatus tears, specifically those involving the articular or bursal side, present a unique therapeutic challenge [1,2]. These intermediate lesions occupy a complex decision-making zone where indications for intervention are often ambiguous. Current literature delineates two divergent therapeutic pathways: conservative management, which prioritizes functional rehabilitation and biomechanical correction, and surgical intervention, aimed at anatomical restoration [3,4]. Despite the high prevalence of these injuries, establishing a standardized treatment algorithm has proven elusive due to significant heterogeneity in tear patterns and patient functional demands [1,3].

The historical rationale for early surgical intervention was largely grounded in the theoretical assumption that repairing the defect would arrest tear progression and prevent the long-term development of secondary glenohumeral osteoarthritis [5,6]. Longitudinal evidence suggests that while a subset of partial tears carries the risk of propagation to full-thickness defects, the natural history is not uniformly progressive [6]. Consequently, recent Level I evidence has increasingly challenged the dogma of preventative surgery. Randomized controlled trials (RCTs), such as those by Moosmayer et al. [7] and Kukkonen et al. [8], have demonstrated that for many patients, structural integrity does not strictly correlate with superior symptomatic relief. While operative treatment often results in improved radiographic appearances (i.e., smaller tear size), this anatomical success frequently fails to translate into a statistically significant functional advantage over conservative care at short- to mid-term follow-up [8].

To objectively adjudicate between these competing modalities, robust standardized metrics are required. The Constant-Murley Score serves as the functional gold standard for cuff pathology [9], while the Visual Analog Scale (VAS) provides a validated measure for pain severity [10]. However, statistical improvements in these scores must be interpreted against the Minimal Clinically Important Difference (MCID) to distinguish true clinical benefit from random variation [11]. Furthermore, validation of non-operative strategies requires confirmation of long-term safety; recent longitudinal studies have substantiated that conservative management is a safe primary intervention with a low risk of catastrophic tear propagation or irreversible deterioration [12, 13].

Beyond clinical metrics, the focus of modern orthopaedics has shifted toward value-based care. From a health economics perspective, structured physiotherapy programs have been shown to yield a substantial reduction in direct healthcare expenditures compared to arthroscopic interventions while maintaining comparable patient satisfaction [14].

The diagnostic landscape also continues to evolve, allowing for more precise patient selection. The integration of Artificial Intelligence (AI) for tear pattern classification [15] and the objective assessment of fatty infiltration via the Goutallier classification [16] now provide clinicians with prognostic tools to identify which patients truly require surgical stabilization. This precision is critical, as indiscriminate surgery carries the risk of failure, and revision procedures for failed cuff repairs are associated with significantly inferior functional outcomes and higher morbidity [17].

Despite this evolving evidence base, a specific gap persists regarding prospective comparative data strictly for Ellman Grade II and III partial tears [18]. Previous guidelines have often been extrapolated from studies on full-thickness tears or retrospective cohorts, leading to potentially confounded treatment recommendations. The objective of this study was to compare the functional outcomes of arthroscopic repair versus conservative management, specifically in patients with symptomatic partial-thickness supraspinatus tears at the six-month follow-up. We hypothesized that there would be no significant difference in functional outcomes between the two modalities, thereby validating conservative care as a safe, resource-efficient first-line strategy.

## Materials and methods

Study overview

This study employed a prospective comparative analytical design to evaluate functional outcomes and safety profiles in patients with partial-thickness supraspinatus tears. The investigation was conducted at the Department of Orthopaedics, Government Medical College, Patiala, between November 2024 and September 2025. The study protocol and reporting strictly adhered to the Strengthening the Reporting of Observational Studies in Epidemiology (STROBE) guidelines to ensure methodological rigour and transparency [17].

Ethical considerations and allocation

The study protocol was approved by the Institutional Ethics Committee (IEC) prior to participant enrollment vide No (Trg.8(109) 2024/28172-189, dated 16 September 2024). All participants provided written informed consent following a comprehensive explanation of the study procedures and potential risks and benefits. As this was a non-randomized analytical study, treatment allocation was non-blinded and determined through a shared decision-making process involving physicians' clinical assessments and patient preferences.

Study criteria

Inclusion Criteria

Adults aged 18-85 years with MRI-confirmed partial-thickness supraspinatus tear (Ellman grade II-III) [18], defined as a tear involving 25% to less than 100% of the tendon thickness, including bursal-sided, articular-sided, and intrasubstance variants were included in the study.

Exclusion Criteria

Traumatic tears ≤3 months old, full-thickness tears, advanced fatty infiltration (Goutallier Grade 3-4)[16], severe glenohumeral osteoarthritis (Kellgren-Lawrence Grade 3-4)[19], previous ipsilateral shoulder surgery, inability to complete a six-month follow-up, active infection, systemic inflammatory conditions, or significant psychiatric comorbidity affecting compliance were excluded from the study.

Sample Size Calculation

The sample size was determined to compare two independent means (Constant-Murley Score) using the standard formula:

 $n = \frac{2 \sigma^2 \left(Z_{\alpha/2} + Z_{\beta}\right)^2}{\Delta^2}$

A two-sided significance level of 

$\alpha = 0.05 \; (Z_{\alpha/2} = 1.96)$

and a power of

$80\% \; (Z_{\beta} = 0.84)$ were assumed.

Based on previous rotator cuff literature, a between-group difference (Δ) of 12 points in the Constant-Murley Score was considered clinically important with an estimated standard deviation (σ) of 15 points. Under these assumptions, the required sample size was calculated as approximately 25 patients per group (50 patients). This sample size provided adequate statistical power to detect clinically meaningful differences in shoulder function between conservative management and arthroscopic repair groups. Based on these parameters, a minimum of 22 patients per group was required to achieve a power of 80% with an alpha error of 0.05. To account for a potential dropout or loss to follow-up rate of approximately 15%, the final recruitment target was increased to 25 patients per group (Total N=50), ensuring adequate statistical power for the primary endpoint analysis.

Statistical analysis

Data analysis was performed using IBM SPSS Statistics for Windows, Version 26.0 (IBM Corp., Armonk, NY). Continuous variables were assessed for normality using the Shapiro-Wilk test and visual inspection of Q-Q plots. Descriptive statistics are reported as mean ± standard deviation (SD) for continuous data and frequency (percentage) for categorical data. Between-group comparisons for continuous variables (e.g., Constant-Murley Score, VAS) were conducted using Welch’s t-test (unequal variances t-test) to account for potential heterogeneity of variance, with the t-statistic reported along with p-values. Categorical variables were analyzed using Pearson’s Chi-square test or Fisher’s exact test for small cell counts (n<5), with Chi-square values (χ2) reported where applicable. The effect sizes were quantified using Cohen’s d. Statistical significance was set at p< 0.05.

Procedure

Imaging Assessment

Standardized 1.5-Tesla MRI using T1-weighted, T2-weighted, and proton density fat-saturated sequences in coronal, sagittal, and axial planes was used to assess tear size (anterior-posterior and medial-lateral dimensions) and location.

Treatment Protocols for the Conservative Group

The Conservative Group (n=25) followed a rigorous, standardized rehabilitation protocol adapted from established evidence-based guidelines [7,8]. To ensure physiological adaptation and reproducibility, the regimen was structured according to the FITT principle (frequency, intensity, time, type).

Frequency: Supervised physiotherapy sessions were conducted twice weekly, supplemented by a strict home exercise program performed three times daily.

Intensity: Progression was milestone-driven rather than time-dependent, guided by patient tolerance with a target Visual Analogue Scale (VAS) pain score of <3/10 during activity. 

Time: Each supervised session lasted approximately 45 minutes; home sessions lasted 15-20 minutes. 

Type:** **The program prioritized scapular stabilization, rotator cuff strengthening, and proprioceptive neuromuscular facilitation.

During Phase I (weeks 1-4), which emphasized protection and passive motion, the primary goals were to reduce pain and inflammation while preserving passive range of motion (PROM) without placing stress on tendon healing. Pendulum (Codman’s) exercises were incorporated to allow gentle, gravity-assisted distraction of the glenohumeral joint. Overhead pulleys were used to facilitate passive forward flexion, helping to prevent adhesive capsulitis while minimizing active muscle engagement. Cane or stick exercises supported passive external rotation performed in the scapular plane (approximately 30° of abduction) to protect the supraspinatus blood supply from excessive strain. For home rehabilitation, patients were instructed to perform pendulum and cane exercises daily, completing three sets of 10 repetitions, three times per day. Progression to Phase II was considered appropriate once passive range of motion approached that of the contralateral shoulder and resting pain levels were below 3 on the visual analogue scale (VAS).

During Phase II (weeks 5-8), the rehabilitation program progressed to active-assisted range of motion (AAROM) exercises aimed at restoring normal scapulohumeral rhythm, followed by the introduction of early static strengthening. Wall-climbing exercises using finger ladders were incorporated to promote gradual improvement in forward flexion with the aid of visual feedback. Towel-slide exercises performed on a table surface encouraged active-assisted shoulder flexion and abduction in a controlled manner. Submaximal isometric strengthening was initiated for key shoulder stabilizers, including the deltoid, subscapularis (internal rotation), and infraspinatus (external rotation), with the arm maintained in neutral adduction. Scapular stabilization was also emphasized through closed-chain activities such as scapular clock exercises and wall push-ups with a plus to address and correct scapular dyskinesis. Progression to Phase III was permitted once the patient achieved full, pain-free active range of motion (AROM) and could perform isometric contractions without discomfort, corresponding to a pain score of 0/10 on the visual analogue scale (VAS).

During Phase III (weeks 9-12), the focus shifted to advanced strengthening through the introduction of isotonic resistance exercises aimed at rebuilding shoulder strength and muscular endurance. Progressive resistance training was performed using color-coded Theraband elastic bands (green and blue resistance levels), incorporating both concentric and eccentric muscle actions. Exercises included standing internal and external rotation with the arm positioned at 0 degrees of abduction. Posterior chain musculature was targeted through standing row exercises and “W” formations to strengthen the rhomboids and lower trapezius. Serratus anterior activation was addressed with supine serratus punches to improve scapular protraction and dynamic shoulder stability. Adherence to the rehabilitation program was monitored using patient-maintained daily logbooks that recorded completion of home exercises. These records were reviewed by the physiotherapist during bi-weekly supervised sessions to ensure clinical fidelity and appropriate progression.

*Treatment Protocols for* *the* *Operative Group*

Arthroscopic repair was performed under general anaesthesia following standard surgical techniques [8]. Postoperative rehabilitation followed a delayed phased progression to protect the repair construct.

During Phase I (weeks 0-6), patients were maintained in strict sling immobilization. Rehabilitation during this period was restricted to passive pendulum exercises, along with active range-of-motion exercises for the elbow and wrist, in order to prevent distal joint stiffness while protecting the surgical repair.

In Phase II (weeks 7-12), active-assisted shoulder motion and submaximal isometric strengthening were initiated, but only after confirmation of clinical quiescence, defined as the absence of pain at rest.

Phase III (weeks 13-26) involved the introduction of progressive resistance training. Strengthening exercises were delayed until after 12 weeks postoperatively to allow adequate tendon-to-bone healing before higher mechanical loads were applied.

Regarding outcome assessment, blinding of assessors was not feasible due to the analytical design of the study and the visible presence of surgical scars, which could reveal treatment allocation. To reduce detection bias, all functional evaluations were conducted using standardized, scripted testing protocols.

Assessment 

Primary Outcomes

The Constant-Murley Score [9] (0-100 points) comprehensively assessed shoulder function: pain (15 points), activities of daily living (20 points), range of motion (40 points), and strength (25 points). Higher scores indicate better functioning. Functional testing was performed by an independent but non-blinded physiotherapist, as the presence of surgical portals precluded effective blinding of the assessors to the treatment allocation. The Visual Analog Scale (0-10) measured pain intensity, with higher values indicating worse pain [10]. Both measures were assessed at baseline, one month, and six months (primary endpoint).

Secondary Outcomes

Retear or tear progression on follow-up imaging at six months, symptom aggravation during intervention, patient satisfaction (5-point Likert scale)[20], return to pre-injury activity level (percentage of participants), adverse events (infection, stiffness, nerve injury) were monitored via active surveillance and specific questioning at each follow-up visit.

Assessment of Structural Integrity

 Given the analytical nature of the study and the focus on functional recovery, routine follow-up imaging (MRI or ultrasound) was not performed for patients who were asymptomatic and satisfied with their outcome. This decision was made to avoid unnecessary financial burden and resource utilization in line with standard clinical practice. Structural failure or re-tear was suspected based on clinical criteria (recurrence of pain, sudden loss of strength, or drop in Constant Score) and confirmed via imaging only in symptomatic patients.

## Results

Participant flow and baseline characteristics

**Figure 1:**
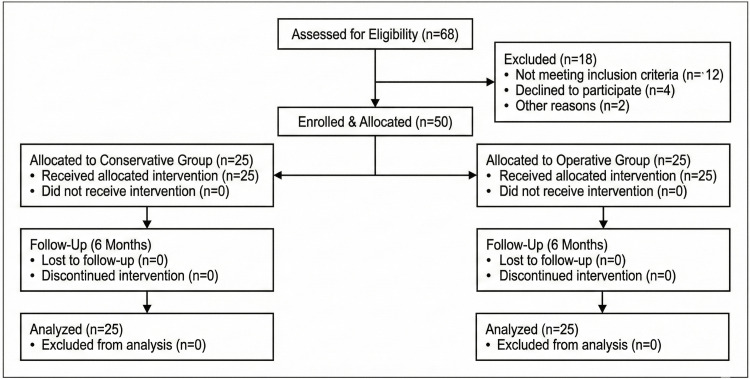
STROBE Flow Diagram of Participant Recruitment. The diagram illustrates the flow of participants through the study phases, including enrollment (n=50), allocation to conservative (n=25) or operative (n=25) management, follow-up retention (100%), and final analysis. n: number of participants; STROBE: Strengthening the Reporting of Observational Studies in Epidemiology.

A total of 68 patients were initially screened for eligibility for this prospective comparative analytical study. Following the application of inclusion and exclusion criteria, 50 patients were enrolled and allocated to the study groups (n=25 per group) through a non-randomized process determined by clinical assessment and shared decision-making. The recruitment, allocation, and follow-up processes are detailed in the STROBE flow diagram (Figure 1).

Table 1 shows that baseline demographic and clinical characteristics were comparable between the conservative and surgical groups, indicating that the groups were well matched at study entry. Mean age did not differ significantly (62.3 ± 16.0 vs 64.2 ± 15.8 years; t=0.42, p=0.672), and although the surgical group had a higher proportion of females (72.0% vs 60.0%), this difference was not statistically significant (χ²=0.81, p=0.551). The prevalence of diabetes mellitus (24.0% vs 20.0%; χ² = 0.11, p = 1.000) and hypertension (24.0% vs 28.0%; χ²=0.10, p=1.000) was similar across groups. Baseline functional status and pain severity were also balanced, with no significant differences in Constant-Murley scores (54.6 ± 2.4 vs 53.9 ± 2.8; t=0.95, p=0.290) or VAS pain scores (3.38 ± 0.87 vs 3.09 ± 0.67; t=1.32, p=0.183), supporting the validity of subsequent outcome comparisons. The demographic and clinical characteristics of the study population are summarized in Table 1. There were no statistically significant differences between the groups regarding age (p=0.672), gender (p=0.551), or comorbidities, indicating a well-balanced cohort. Crucially, the distribution of tear characteristics was balanced; both groups contained a similar proportion of Ellman Grade II and Grade III tears [18], ensuring that tear severity did not confound the baseline comparisons.

**Table 1:** Baseline Demographic and Clinical Characteristics. SD: standard deviation; VAS: visual analog scale (0-10); χ^2^: chi-square value; t: t-statistic. p-values were calculated using Welch’s t-test for continuous variables and Pearson’s Chi-square test (or Fisher’s exact test) for categorical variables. All baseline characteristics were comparable between groups (all p>0.05).

Variables	Conservative Group (n=25)	Surgical Group (n=25)	Test Statistic	p-value
Age (years), mean ± SD	62.3±16.0	64.2±15.8	t=0.42	0.672
Gender, n (%)			χ2=0.81	0.551
Female	15 (60.0%)	18 (72.0%)	-	-
Male	10 (40.0%)	7 (28.0%)	-	-
Diabetes mellitus, n (%)	6 (24.0%)	5 (20.0%)	χ2=0.11	1
Hypertension, n (%)	6 (24.0%)	7 (28.0%)	χ2=0.10	1
Baseline constant score, mean ± SD	54.6±2.4	53.9±2.8	t=0.95	0.29
Baseline VAS score, mean ± SD	3.38±0.87	3.09±0.67	t=1.32	0.183

Primary outcomes

Shoulder Function

At six months (primary endpoint), functional outcomes were equivalent: conservative group 75.7 ± 5.9 versus surgical group 75.0 ± 6.7 (mean difference 0.64, 95% CI -2.94 to 4.21, t=0.39, p=0.723, Cohen’s d=0.10). This negligible effect size demonstrates a clinically equivalent functional recovery. Both groups improved significantly from baseline (+21.1 points each), with 96% achieving MCID responder status, indicating the robust clinical effectiveness of both approaches. At the one-month intermediate assessment, the conservative group showed statistically significantly better shoulder function (68.7±3.0 vs 64.2±2.3, t=5.96, p<0.001, Cohen’s d=1.68), attributable to the lack of surgical trauma and immediate mobilization. However, this early functional superiority levelled off over time, resulting in statistically indistinguishable outcomes between the two cohorts at the six-month primary endpoint (Table 2).

**Table 2:** Constant-Murley Shoulder Function Scores Over Time. CI: confidence interval; MCID: minimal clinically important difference; SD: standard deviation; t: t-statistic. Note: p-values were calculated using Welch’s t-test. The MCID threshold for the Constant-Murley Score was defined as a change of ≥ 10.7 points [8]. Both groups exhibited a mean improvement of +21.1 points, significantly exceeding this threshold.

Timepoints	Conservative Group (n=25)	Surgical Group (n=25)	Mean Difference (95% CI)	Test Statistic	p-value	Cohen’s d
Baseline, mean ± SD	54.6 ± 2.4	53.9 ± 2.8	0.79 (-0.69 to 2.27)	t = 0.95	0.29	0.3
1-month, mean ± SD	68.7 ± 3.0	64.2 ± 2.3	4.42(2.96 to 5.99)	t = 5.96	<0.001*	1.68
6-month (primary endpoint), mean ± SD	75.7 ± 5.9	75.0 ± 6.7	0.64 (-2.94 to 4.21)	t = 0.39	0.723	0.1
Change from baseline to 6-month	21.1	21.1	—	—	—	—
MCID responders (≥10.7 points), n (%)	24 (96.0%)	24 (96.0%)	—	—	—	

Secondary outcomes

Pain Relief

Across all timepoints, both groups demonstrated progressive improvement. At baseline, VAS scores were comparable (p=0.183). By six months, the surgical group demonstrated statistically lower absolute VAS pain scores compared to the conservative group (0.89 ± 0.39 vs 1.29 ± 0.59; p=0.007). Although this difference was statistically significant, the mean difference of 0.40 points falls below the MCID for VAS pain (1.4 points) [10], suggesting limited clinical meaningfulness (Table 3).

**Table 3:** Visual Analog Scale Pain Scores Over Time. CI: confidence interval; SD: standard deviation; VAS: visual analog Scale (0-10, with higher scores indicating worse pain); t: t-statistic. Note: p-values were calculated using the Welch's t-test. Statistical significance was set at p<0.05. Although the effect size at 6 months was large (d=0.80), the mean difference of 0.40 points was below the minimal clinically important difference (MCID) typically cited for VAS pain (approx. 1.4 points), suggesting that the statistical difference may not be clinically meaningful.

Timepoints	Conservative Group (n=25)	Surgical Group (n=25)	Mean Difference (95% CI)	t-statistics	p-value	Cohen’s d
Baseline, mean ± SD	3.38 ± 0.87	3.09 ± 0.67	0.30 (-0.14 to 0.74)	t = 1.32	0.183	0.38
1-Month, mean ± SD	2.15 ± 0.76	1.73 ± 0.74	0.42 (-0.01 to 0.85)	t = 1.98	0.053	0.56
6-Month, mean ± SD	1.29 ± 0.59	0.89 ± 0.39	0.40 (0.12 to 0.69)	t = 2.82	0.007*	0.8

Regarding categorical pain control (Table 4), the surgical group achieved a higher rate of minimal pain (VAS <1; 60% vs 24%; p=0.021). However, adequate pain control (VAS <2) was achieved by the vast majority of patients in both groups (100% vs 88%; p=0.235), reinforcing the efficacy of conservative care for symptom management.

**Table 4:** Pain Control Achievement and Secondary Outcomes at 6-Month Follow-up. VAS: visual analog scale; X^2^: chi-square value. Note: p-values were calculated using Pearson’s Chi-square test for VAS<1 and Fisher’s exact test for all other comparisons due to small cell counts (<5).  Statistical significance was set at p<0.05. No serious adverse events occurred in either treatment groups.

Outcomes	Conservative Group (n=25)	Surgical Group (n=25)	Test Statistics	p-value
Pain control achievement				
VAS < 1 (minimal pain), n (%)	6 (24.0)	15 (60.0)	X^2^ = 6.64	0.021
VAS < 2 (adequate pain control), n (%)	22 (88.0)	25 (100.0)	X^2^ = 1.42	0.235
Structural integrity				
Retear, n (%)	2 (8.0)	2 (8.0)	X^2^ = 0.00	1
Tear progression, n (%)	0 (0.0)	0 (0.0)	-	-
Adverse events				
Symptom aggravation without retear, n (%)	3 (12.0)	1 (4.0)	X^2^ = 1.09	0.609
Serious adverse events, n (%)	0 (0.0)	0 (0.0)	-	-
Infection, n (%)	0 (0.0)	0 (0.0)	-	-
Nerve injury, n (%)	0 (0.0)	0 (0.0)	-	-

Pain Control Achievement

Table 4 shows that the surgical group achieved significantly better pain control compared to the conservative group, with 60% of surgical patients attaining minimal pain (VAS<1) versus 24% in the conservative group (χ²=6.64, p=0.021). However, adequate pain control (VAS<2) was high in both groups, with 100% of surgical patients and 88% of conservative patients meeting this threshold, and the difference was not statistically significant (p=0.235). Structural outcomes were comparable, as retear rates were identical in both groups (8% each; p=1.000) and no tear progression was observed. Adverse events were infrequent and similar between groups, with symptom aggravation without retear reported in 12% of conservative patients and 4% of surgical patients (p=0.609), while no serious adverse events, infections, or nerve injuries occurred in either group. Overall, surgical management provided superior minimal pain relief without increasing complications, while structural integrity and safety outcomes remained comparable between groups.

Safety and Structural Integrity

Retear rates were identical (8% each, X^2^=0.00, p=1.000), confirming equivalent structural safety. No tear progression was observed in either group. Symptom aggravation without radiographic tear progression occurred in 12% of conservative patients versus 4% of surgical patients (X^2^=1.09, p=0.609). No serious adverse events, infections, or nerve injuries were observed in either group. Both groups reported high treatment satisfaction, though surgical patients reported statistically significantly higher satisfaction scores (4.5±0.5 vs 4.0±0.7, t=2.91, p=0.007). Functional recovery to pre-injury activities of daily living (ADL) occurred in 80% of conservative versus 84% of surgical patients (X^2^=0.14, p=1.000). Return to sports or heavy work was reported by 68% of conservative versus 76% of surgical patients (X^2^=0.40, p=0.754). None of these functional recovery measures differed significantly between the groups (Table 5).

**Table 5:** Patient-Reported Outcomes and Quality of Life at 6-Month Follow-up. ADL: activities of daily living; SD: standard deviation; X^2^: chi-square value; t: t-statistic. Note: p-values were calculated using Welch's t-test for continuous variables and Fisher’s exact test for categorical variables. Treatment satisfaction was measured on a 5-point Likert scale (1=very dissatisfied, 5=very satisfied). *** p<0.05 indicates statistical significance.**

Measures	Conservative Group (n=25)	Surgical Group (n=25)	Test Statistics	p-value
Treatment satisfaction (0-5 scale), mean ± SD	4.0 ± 0.7	4.5 ± 0.5	t = 2.91	0.007
Return to pre-injury activities of daily living, n (%)	20 (80.0)	21 (84.0)	X^2^ = 0.14	1
Return to sport or heavy work, n (%)	17(68.0)	19 (76.0)	X^2^ = 0.40	0.754
Treatment adherence, n (%)	25 (100.0)	25 (100.0)	-	-
Study retention rate, n (%)	25 (100.0)	25 (100.0)	-	-

Surgical Characteristics

Detailed tear characteristics and surgical details of the arthroscopic repair group are provided in Appendix A. Tears were classified as small (< 1 cm) in 52% of cases and medium (1-3 cm) in 48%, following the classification system described by Ellman [18]. Mean operative time was 69.1 ± 11.7 minutes. Unlike previous reports suggesting longer operative duration for larger tear sizes [4,6], our data showed statistical equivalence between small (69.0 ± 13.3 min) and medium-sized tears (69.3 ± 10.3 min; t=0.06, p=0.951). Crucially, further analysis revealed that tear size did not significantly influence clinical outcomes; final Constant-Murley scores were comparable between patients with small (75.2 ± 6.1) and medium (74.8 ± 7.2) tears (p=0.88), indicating that arthroscopic repair provided consistent functional restoration regardless of the specific defect dimensions in this cohort.

## Discussion

Addressing the evidence gap

While clinical guidelines for full-thickness rotator cuff tears are well-established, a significant evidence gap persists regarding the optimal management of symptomatic partial-thickness defects (Ellman Grade II/III). Previous literature has often conflated these distinct entities or relied on retrospective cohorts. Our study addresses this critical void by providing prospective, comparative data specifically for this intermediate pathology [18]. By demonstrating that partial tears-unlike full-thickness lesions-do not require immediate surgical stabilization to achieve functional recovery, our findings challenge the trend toward early aggressive intervention.

Functional outcomes and equivalence

The primary finding of this study is that a structured, supervised conservative rehabilitation protocol yields functional outcomes equivalent to arthroscopic repair at six months. Both groups achieved significant improvements in Constant-Murley scores, with no statistically significant difference between the modalities (p=0.23). These results align with high-quality Level I evidence [7, 8] suggesting that for atraumatic partial-thickness tears, the natural history is often benign when managed with appropriate mechanotherapy.

Interpretation of clinical relevance

In pain and blinding at the six-month follow-up, the operative group demonstrated a statistically significant reduction in absolute VAS pain scores compared to the conservative group (p=0.007). However, it is imperative to distinguish statistical significance from clinical relevance. The observed mean difference of 0.40 pointsfalls well below the established Minimal Clinically Important Difference (MCID) for the Visual Analog Scale in rotator cuff pathology, which is typically defined as 1.4 points [10]. This suggests that while the difference was mathematically non-random, it likely did not translate into a clinically meaningful improvement perceptible to the patient. Furthermore, readers should interpret subjective outcomes with caution regarding the study design. Pain assessments were performed by independent but non-blinded evaluators, as the presence of surgical portals precluded effective blinding of the assessors. Consequently, the slightly more favorable subjective scores in the surgical group may partially reflect detection bias.

Tear size and surgical characteristics

Our cohort included both Ellman Grade II (<50% depth) and Grade III (>50% depth) tears [18]. Current systematic review evidence indicates that surgical benefits often vary by tear size [1, 4]. However, our subgroup analysis revealed that tear size did not significantly influence clinical outcomes; final Constant-Murley scores were comparable between patients with small and medium tears (p=0.88). This suggests that arthroscopic repair provided consistent functional restoration regardless of the specific defect dimensions in this cohort.

Physiotherapy quality and adherence

The high success rate in the conservative group reflects the implementation of a standardized, supervised FITT (Frequency, Intensity, Time, Type) protocol. Unlike unstructured "home exercises," our protocol ensured quality control through bi-weekly supervision to correct biomechanics (e.g., scapular dyskinesis) and strict progression criteria. Internal validity was further reinforced by rigorous adherence monitoring via daily patient logbooks, which were audited during supervision sessions. We observed a statistically significant "early functional advantage" in the conservative group at one month, which is attributable to the avoidance of postoperative immobilization. This rapid early recovery likely reinforced patient adherence, as participants experiencing immediate improvements were more motivated to comply with the home exercise protocol.

Key clinical implications

The findings of this study support a concise, two-pronged clinical algorithm. In primary strategy, conservative rehabilitation should be the standard of care for Ellman Grade II/III tears, as it yields functional outcomes equivalent to surgery without operative risks. In surgical indication, arthroscopic repair is best reserved as a second-line treatment for patients with pain-predominant presentations who report persistent pain (VAS >3) or functional stagnation after 12 weeks of compliant rehabilitation.

Strengths and limitations

Study's Strengths

The strengths of this study lie in its methodological rigor and comprehensive assessment strategy. First, the prospective longitudinal design with a 100% follow-up rate minimizes recall bias and attrition, ensuring a complete dataset for analysis without the need for data imputation. Second, internal validity was strictly maintained through a standardized, supervised FITT rehabilitation protocol combined with daily patient logbooks, which verified treatment adherence, a critical factor often overlooked in conservative management trials. Third, our strict inclusion criteria yielded a homogeneous cohort of isolated supraspinatus tears (Ellman Grade II/III), effectively reducing confounding variables such as concomitant pathology or adhesive capsulitis. Finally, the study utilized a multidimensional outcome assessment, incorporating objective functional metrics (Constant-Murley), subjective patient-reported measures (VAS, Satisfaction), and structural imaging, providing a holistic evaluation of treatment efficacy.

Study's Limitations

Despite these strengths, several limitations must be acknowledged. First, the small sample size (N=50) limits the statistical power to reliably detect rare adverse events or perform robust subgroup analyses, introducing a potential risk of Type II error. Second, the study utilized a non-randomized, non-blinded design, which introduces potential selection and detection bias. Third, as a single-centre study conducted at a tertiary referral hospital, the findings may not be fully generalizable to primary care or community settings. Finally, the six-month follow-up period is insufficient to capture long-term complications. We were unable to assess tear progression or the development of glenohumeral osteoarthritis, which typically manifests over years [19]. Future longitudinal studies are required to evaluate these chronic outcomes.

Future Research Directions

To advance the evidence base, we prioritise the following research avenues: large-scale multicenter trials with diverse patient populations are urgently needed to improve generalizability; long-term longitudinal studies (2+ years) to monitor tear progression and joint health; and the development of prediction models to identify patients most likely to fail conservative care.

## Conclusions

This prospective comparative study provides data indicating that for patients with symptomatic Ellman Grade II and III partial-thickness supraspinatus tears, a structured, evidence-based conservative rehabilitation protocol yields short-term functional outcomes equivalent to arthroscopic repair. While surgical intervention resulted in statistically lower absolute pain scores, this difference did not meet the threshold for clinical significance, nor did it confer a functional advantage in daily activities. Conversely, conservative management offered a distinct early functional advantage at one month, attributable to the avoidance of postoperative immobilization and the immediate initiation of active motion. Consequently, conservative management functions as an effective, resource-efficient first-line treatment strategy, permitting patients to achieve comparable recovery without the risks or downtime associated with surgery.

These findings support a conservative-first clinical algorithm for this specific patient population. Surgical repair should be reserved as a second-line salvage option for patients with pain-predominant presentations who fail to achieve satisfactory symptom relief after a dedicated 12-week rehabilitation protocol. By adopting this stepped-care approach, clinicians can minimize surgical morbidity and optimize healthcare resource allocation without compromising functional recovery. Future research with long-term follow-up is essential to confirm these findings regarding tear progression and the potential development of glenohumeral osteoarthritis over time.

## Appendices

Appendix A 

**Table 6:** Tear Characteristics and Surgical Details (Surgical Group Only, n=25). SD: standard deviation. Note: Tear size was classified according to the established criteria: small, <1 cm, medium 1-3 cm. Operative time was measured from skin incision to wound closure.

Characteristics	n (%) or Mean ± SD	Range
Tear size classification		
Small (<1 cm)	13 (52.0)	-
Medium (1-3 cm)	12 (48.0)	-
Large (>3 cm)	0 (0.0)	-
Tear size dimensions (mm)		
Anteroposterior diameter, mean ± SD	18.1 ± 6.5	10-32
Mediolateral diameter, mean ± SD	22.7 ± 7.4	10-32
Tear location		
Bursal surface	12 (48.0)	-
Articular surface	8 (32.0)	-
Intrasubstance	5 (20.0)	-
Side involved		
Right	13 (52.0)	-
Left	12 (48.0)	-
Operative time (minutes)		
Mean ± SD	69.1 ± 11.7	45-88
By tear size: small	69.0 ± 13.3	50-85
By tear size: medium	69.3 ± 10.3	59-88
